# Supplementary material for: Association between non-acute Traumatic Injury (TI) and Heart Rate Variability (HRV) in adults: A systematic review and meta-analysis
Source: PLoS One. 2023 Jan 23;18(1):e0280718. doi: 10.1371/journal.pone.0280718 (PMC9870143; doi:10.1371/journal.pone.0280718)
Supplement: S1 Fig — Funnel plot for outcomes SDNN (A), RMSSD (B) and LF: HF ratio (C). (DOCX) [file pone.0280718.s002.docx]

**Supporting Information 8: Funnel plot for outcomes SDNN (A), RMSSD (B) and LF: HF ratio (C)**


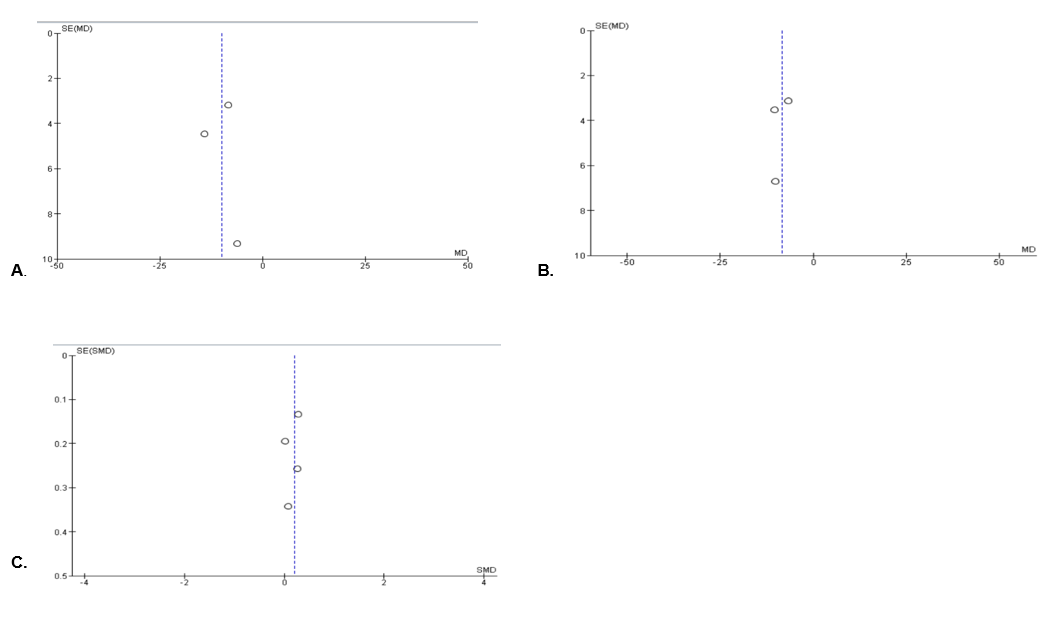
Abbreviations: standard deviation of NN intervals (SDNN), the root mean square of successive differences between normal heartbeats (RMSSD), Low Frequency (LF), High Frequency (HF)
