# Supplementary material for: Association between non-acute Traumatic Injury (TI) and Heart Rate Variability (HRV) in adults: A systematic review and meta-analysis
Source: PLoS One. 2023 Jan 23;18(1):e0280718. doi: 10.1371/journal.pone.0280718 (PMC9870143; doi:10.1371/journal.pone.0280718)
Supplement: S2 Table — (DOCX) [file pone.0280718.s004.docx]

**Supporting information 3: The Inclusion and Exclusion criteria**

|  | **Inclusion** | **Exclusion** |
| --- | --- | --- |
| **Population** | Young/middle/older adults from both sexes, aged >18, no history of cardiovascular disease | Animals, children, or adolescents aged <18, history of cardiovascular disease |
| **Exposure** | any physical trauma sustained (>7 days post injury) such  as gunshot wounds, amputation, limb loss, burn and fall etc. | Studies which involve PTSD, depression, anxiety, head/brain injury, traumatic brain injury, spinal cord injury and haemorrhage. |
| **Comparison** | Controls with no traumatic injury. | No control |
| **Outcome** | Any index of HRV | No HRV measure reported as a primary of secondary outcome. |
| **Setting** | Any | **-** |
| **Study Design** | Observational, cohort, cross-sectional, prospective, case control | Systematic reviews, reviews |
| **Language** | English | Other than English |
| **Date** | Any | - |
| **Publication status** | Published research papers | In-press, grey literature, conference proceedings, meeting abstracts, case reports, case series, opinion/editorial, in-vitro and animal study. |

Abbreviations: Post-traumatic stress disorder (PTSD), Heart Rate Variability (HRV)
