# Supplementary material for: Association between non-acute Traumatic Injury (TI) and Heart Rate Variability (HRV) in adults: A systematic review and meta-analysis
Source: PLoS One. 2023 Jan 23;18(1):e0280718. doi: 10.1371/journal.pone.0280718 (PMC9870143; doi:10.1371/journal.pone.0280718)
Supplement: S4 Table — (DOCX) [file pone.0280718.s006.docx]

**Supporting information 5: The data extraction form**

|  | **Item to be extracted** | **Data** | **Location (Pg. no)** |
| --- | --- | --- | --- |
| **General information** | Data of data extraction |  |  |
|  | Date of form completed |  |  |
|  | Name of reviewer who extracted data |  |  |
| **General study information** | Study ID |  |  |
|  | Author, date of publication |  |  |
|  | Title |  |  |
|  | Type of publication |  |  |
| **Study characteristics** | Study type (research or review) |  |  |
|  | Aims |  |  |
|  | Sample size at the start of the study |  |  |
|  | Sample size included in the analysis |  |  |
|  | Participant’s characteristics |  |  |
|  | Participants’ gender |  |  |
|  | Participants’ age |  |  |
|  | Setting (Country, location) |  |  |
| **Methods** | Study timeline |  |  |
|  | Study design |  |  |
|  | Recruitment (sampling technique) |  |  |
|  | Consent of participants |  |  |
|  | Inclusion criteria |  |  |
|  | Exclusion criteria |  |  |
|  | Exposure variable (or type of injury) |  |  |
|  | Measurement of exposure variable |  |  |
|  | Time from injury |  |  |
|  | Primary outcome variable (HRV) |  |  |
|  | Measurement of primary outcome variable (HRV) |  |  |
|  | Posture while HRV measurement |  |  |
|  | Secondary outcome variable (HR) and measurement |  |  |
|  | Comparator group size |  |  |
|  | Comparator group characteristics |  |  |
| **Results** | Software for HRV analysis |  |  |
|  | Missing data |  |  |
|  | Data analysis methods |  |  |
|  | Result type (means, SD, correlation coefficients, risk ratio, odds ratio etc). |  |  |
|  | Results (primary outcome) HRV |  |  |
|  | Results (secondary outcome) HR |  |  |
|  | Results (adjusted analyses) |  |  |
| **Strengths and weakness** | Strengths |  |  |
|  | Limitations |  |  |
|  | Conflict of interest |  |  |

Abbreviations: Heart Rate (HR), Heart Rate Variability (HRV)
