# Supplementary material for: Association between non-acute Traumatic Injury (TI) and Heart Rate Variability (HRV) in adults: A systematic review and meta-analysis
Source: PLoS One. 2023 Jan 23;18(1):e0280718. doi: 10.1371/journal.pone.0280718 (PMC9870143; doi:10.1371/journal.pone.0280718)
Supplement: S5 Table — (DOCX) [file pone.0280718.s007.docx]

**Supporting information 6: The Axis Quality Appraisal score for the included studies**

|  | **Year** | **1** | **2** | **3** | **4** | **5** | **6** | **7** | **8** | **9** | **10** | **11** | **12** | **13** | **14** | **15** | **16** | **17** | **18** | **19** | **20** | **Total** |
| --- | --- | --- | --- | --- | --- | --- | --- | --- | --- | --- | --- | --- | --- | --- | --- | --- | --- | --- | --- | --- | --- | --- |
| Peles et al. | 1995 | 1 | 1 | 0 | 1 | 1 | 0 | 0 | 1 | 1 | 0 | 1 | 0 | 1 | 0 | 1 | 0 | 1 | 0 | 0 | 1 | 11 |
| De Kooning et al. | 2013 | 1 | 1 | 1 | 1 | 1 | 1 | 0 | 1 | 1 | 0 | 1 | 0 | 1 | 0 | 1 | 0 | 1 | 1 | 0 | 1 | 14 |
| Joo et al. | 2017 | 1 | 1 | 0 | 1 | 1 | 0 | 0 | 0 | 1 | 1 | 1 | 1 | 1 | 0 | 1 | 1 | 1 | 1 | 0 | 1 | 14 |
| Pozzato et al. | 2021 | 1 | 1 | 1 | 1 | 1 | 1 | 1 | 1 | 1 | 0 | 1 | 1 | 1 | 1 | 1 | 1 | 1 | 1 | 1 | 1 | 19 |

1.     Were the aims/objectives of the study clear?

2.       Was the study design appropriate for the stated aims?

3.       Was the sample size justified?

4.       Was the target/reference population clearly defined? (Is it clear who the research was about?)

5.       Was the sample frame taken from an appropriate population base so that it closely represented the target/reference population under investigation?

6.       Was the selection process likely to select subjects /participants that were representative of the target/ reference population under investigation?

7.       Were measures undertaken to address and categorize non-responders?

8.       Were the risk factor and outcome variables measured appropriate to the aims of the study?

9.       Were the risk factor and outcome variables measured correctly using instruments/ measurements that had been trialled, piloted, or published previously?

10.     Is it clear what was used to determined statistical significance and/or precision estimates? (e.g., p values, CIs)

11.     Were the methods (including statistical methods) sufficiently described to enable them to be repeated?

12.     Were the basic data adequately described?

13.     Does the response rate not raise concerns about non- response bias?

14.     If appropriate, was information about non- responders described?

15.     Were the results internally consistent?

16.     Were the results for the analyses described in the methods presented?

17.     Were the authors’ discussions and conclusions justified by the results?

18.     Were the limitations of the study discussed?

19.     Were there no funding sources or conflicts of interest that may affect the authors’ interpretation of the results?

20.     Was ethical approval or consent of participants attained?
