# Supplementary material for: Association between non-acute Traumatic Injury (TI) and Heart Rate Variability (HRV) in adults: A systematic review and meta-analysis
Source: PLoS One. 2023 Jan 23;18(1):e0280718. doi: 10.1371/journal.pone.0280718 (PMC9870143; doi:10.1371/journal.pone.0280718)
Supplement: S6 Table — (DOCX) [file pone.0280718.s008.docx]

**Supporting information 7: The Risk of Bias score of each study using Risk of Bias Assessment tool for Non-randomized Studies**

| **Study** | **Selection of participants** | **Confounding Variables** | **Exposure Measurement** | **Blinding of Outcome Assessment** | **Incomplete Outcome data** | **Selective Outcome reporting** | **Overall Bias Risk (0-6)** |
| --- | --- | --- | --- | --- | --- | --- | --- |
| Peles et al. 1995 | 1 | 0 | 0 | 1 | 1 | 1 | 4 |
| De Kooning et al. 2013 | 0 | 0 | 1 | 1 | 1 | 1 | 4 |
| Joo et al. 2018 | 1 | 1 | 0 | 1 | 0 | 0 | 3 |
| Pozzato et al.2021 | 0 | 0 | 0 | 1 | 0 | 0 | 1 |

Note: Risk of bias rated as low=0, high =1 and – means results unclear
