# Supplementary material for: Association between non-acute Traumatic Injury (TI) and Heart Rate Variability (HRV) in adults: A systematic review and meta-analysis
Source: PLoS One. 2023 Jan 23;18(1):e0280718. doi: 10.1371/journal.pone.0280718 (PMC9870143; doi:10.1371/journal.pone.0280718)
Supplement: S7 Table — (DOCX) [file pone.0280718.s009.docx]

**Supporting Information 9: GRADE Evidence Profile (a) and summary of findings table (b) of Outcome Measures SDNN, RMSSD and LF: HF ratio.**

| 1. **GRADE Evidence Profile** | | | | | | | | | | | |
| --- | --- | --- | --- | --- | --- | --- | --- | --- | --- | --- | --- |
| **Certainty assessment** | | | | | | | **№ of participants** | | **Effect** | **Certainty** | **Importance** |
| **№ of studies** | **Study design** | **Risk of bias** | **Inconsistency** | **Indirectness** | **Imprecision** | **Other considerations** | **Injured** | **Uninjured** | **Standardised Mean Difference (SMD)**  **Mean Difference (MD)** |  |  |
| **SDNN** | | | | | | | | | | | |
| 3 | observational studies | serious^a^ | not serious^b^ | not serious^c^ | not serious^d^ | strong association | 206 | 150 | MD **9.93 lower** (14.82 lower to 5.03 lower) | ⨁⨁◯◯ Low | IMPORTANT |
| **RMSSD** | | | | | | | | | | | |
| 3 | observational studies | serious^a^ | not serious^b^ | not serious^c^ | not serious^d^ | strong association | 206 | 150 | MD **8.45 ms lower** (12.78 lower to 4.12 lower) | ⨁⨁◯◯ Low | IMPORTANT |
| **LF/HF** | | | | | | | | | | | |
| 4 | observational studies | serious^a^ | not serious^e^ | not serious^f^ | serious^g^ | none | 258 | 203 | SMD **0.2 SD higher** (0.01 higher to 0.39 higher) | ⨁◯◯◯ Very low | IMPORTANT |

| 1. **GRADE summary of Evidence** | | | | |
| --- | --- | --- | --- | --- |
| **Outcomes** | **Anticipated absolute effects^*^ (95% CI)** | **№ of participants (studies)** | **Certainty of the evidence (GRADE)** | **Comments** |
|  | **Mean difference (MD), Standardised mean difference (SMD)** |  |  |  |
| SDNN | MD **9.93 lower** (14.82 lower to 5.03 lower) | 356 (3 observational studies) | ⨁⨁◯◯ Low^a,b,c,d^ | The evidence suggests that people who sustained traumatic injury may have lower SDNN (ms) as compared to people with no injury. |
| RMSSD | MD **8.45 ms lower** (12.78 lower to 4.12 lower) | 356 (3 observational studies) | ⨁⨁◯◯ Low^a,b,c,d^ | The evidence suggests that people who sustained traumatic injury may have lower RMSSD (ms) as compared to people with no injury. |
| LF/HF | SMD **0.2 SD higher** (0.01 higher to 0.39 higher) | 461 (4 observational studies) | ⨁◯◯◯ Very low^a,e,f,g^ | People with a non-acute TI may have a slightly higher LF/HF ratio than those without the injury- which represents autonomic imbalance. |

#### Explanation:

#### no blinding done at any point, selective reporting of outcome data in 1 study, different method of HRV measurements and recording times

#### the trend of results is similar across all 3 studies despite difference in methods

#### PECO clearly defined in all 3 papers

#### Not explicitly described in 2 papers but mentioned.

#### the trend of results is similar across all 4 studies despite difference in methods

#### PECO clearly defined in all 4 papers

#### Two studies had not reported the data in the paper. The authors of de Kooning et al. 2013 study were contacted. Data was approximated through graphs presented in Peles et al. 1995 study as the contact was not possible.

Note: The scoring of each outcome was downgraded one level if:

1. High risk of bias identified in 1 or 2 studies due to inappropriate sampling, and selective reporting (Axis questions: 3, 7, 14, 12, 16)
2. Imprecision identified in 1 or 2 studies due to poor statistical analysis and lack of sample size justification (Axis questions 3 and 10).
3. Indirectness identified in 1 or 2 studies due to research questions and outcomes not according to PECO (Axis question 4).
4. Inconsistency identified in 1 or 2 studies due to heterogeneity in results.

#### Abbreviations: standard deviation of NN intervals (SDNN), the root mean square of successive differences between normal heartbeats (RMSSD), Low Frequency (LF), High Frequency (HF), confidence interval (CI), mean difference (MD), standardized mean difference (SMD).
