# Supplementary material for: Association between non-acute Traumatic Injury (TI) and Heart Rate Variability (HRV) in adults: A systematic review and meta-analysis
Source: PLoS One. 2023 Jan 23;18(1):e0280718. doi: 10.1371/journal.pone.0280718 (PMC9870143; doi:10.1371/journal.pone.0280718)
Supplement: Attachment — Submitted filename: Response to Reviewers.docx [file pone.0280718.s010.docx]

**PONE-D-22-26222**

**Association between non-acute Traumatic Injury (TI) and Heart Rate Variability (HRV) in adults: a systematic review and meta-analysis**

We would like to thank the academic editor and the reviewer for providing insightful feedback on our manuscript. The suggestions made by the academic editor and the reviewer have been incorporated into the manuscript and are highlighted. Please see below, in blue, for a point-by-point response to the reviewer’s comments. We hope that this manuscript might now be considered suitable for publication.

**Journal Requirements:**

When submitting your revision, we need you to address these additional requirements.

1. Please ensure that your manuscript meets PLOS ONE's style requirements, including those for file naming. The PLOS ONE style templates can be found at
   <https://journals.plos.org/plosone/s/file?id=wjVg/PLOSOne_formatting_sample_main_body.pdf> and 
   <https://journals.plos.org/plosone/s/file?id=ba62/PLOSOne_formatting_sample_title_authors_affiliations.pdf>

Response: Thank you for bringing this to our attention. I have checked the format and –to the best of our knowledge- the manuscript has now been revised and written according to PLOS One’s style requirements, including those for file naming, authors’ affiliations, and supporting information. The figure captions have also been added in the manuscript (Line 203 & Lines 271-276 and figures have been uploaded in TIFF format, as per PLOS One’s format requirement.

2. Thank you for stating in your Funding Statement: 
"This project is a part of RM’s Ph.D. studentship- jointly funded by Bournemouth University and the ADVANCE charity, UK."

Please provide an amended statement that declares *all* the funding or sources of support (whether external or internal to your organization) received during this study, as detailed online in our guide for authors at <http://journals.plos.org/plosone/s/submit-now>.  Please also include the statement “There was no additional external funding received for this study.” in your updated Funding Statement.

Response: Thank you for this comment. The revised funding statement has been stated in the cover letter as recommended by the journal:

*This project is a part of RM’s Ph.D. studentship- jointly funded by Bournemouth University and the ADVANCE charity, UK. The funders had no role in study design, data collection and analysis, decision to publish, or preparation of the manuscript. There was no additional external funding received for this study.*

Please include your amended Funding Statement within your cover letter. We will change the online submission form on your behalf.

Response: Noted. The Funding Statement has now been amended with the cover letter.

3. Thank you for stating the following financial disclosure: 
"This project is a part of RM’s Ph.D. studentship- jointly funded by Bournemouth University and the ADVANCE charity, UK."

Please state what role the funders took in the study. If the funders had no role, please state: ""The funders had no role in study design, data collection and analysis, decision to publish, or preparation of the manuscript."" 
If this statement is not correct you must amend it as needed.

Response: Thanks for highlighting this. The revised funding statement has been mentioned in the cover letter. Please see the response to comment 2.

Please include this amended Role of Funder statement in your cover letter; we will change the online submission form on your behalf.

Response: Thank you, the Role of Funder has now been amended in the cover letter.

4. In your Data Availability statement, you have not specified where the minimal data set underlying the results described in your manuscript can be found. PLOS defines a study's minimal data set as the underlying data used to reach the conclusions drawn in the manuscript and any additional data required to replicate the reported study findings in their entirety. All PLOS journals require that the minimal data set be made fully available. For more information about our data policy, please see <http://journals.plos.org/plosone/s/data-availability>.

Upon re-submitting your revised manuscript, please upload your study’s minimal underlying data set as either Supporting Information files or to a stable, public repository and include the relevant URLs, DOIs, or accession numbers within your revised cover letter. For a list of acceptable repositories, please see <http://journals.plos.org/plosone/s/data-availability#loc-recommended-repositories>. Any potentially identifying patient information must be fully anonymized.

Response: Thank you for this suggestion. However, this is a systematic review of existing evidence. No new dataset was generated. The data (mean differences/ standardised mean difference between injured and control groups) were extracted from the included studies and pooled in RevMan. The studies included in the systematic review have been cited and referenced in the manuscript- the readers can access those studies to access original data if needed.

Please note the data extraction forms and the data resulting from the critical appraisal, risk of bias assessment, and strength of evidence have been uploaded as supporting material and are available for the readers.

Important: If there are ethical or legal restrictions to sharing your data publicly, please explain these restrictions in detail. Please see our guidelines for more information on what we consider unacceptable restrictions to publicly sharing data: <http://journals.plos.org/plosone/s/data-availability#loc-unacceptable-data-access-restrictions>. Note that it is not acceptable for the authors to be the sole named individuals responsible for ensuring data access.

Response: Not applicable.

**Review Comments to the Author:**

Reviewer #1: Rabeea et al. conducted a study on "Association between Non-Acute Traumatic Injury (TI) and Heart Rate Variability (HRV) in Adults: A Systematic Review and Meta-analysis,"  in which they investigated the impact of non-acute TI on standard HRV indices in adults. This study can be improved by incorporating the following points:
 
In the introduction, the authors should highlight how this study addresses a current gap in the literature..

Response: Thank you for this comment. We would like to draw your attention to the following section in the Introduction which answers this question- how does this study address a current gap in the literature?

Lines 81-88: *“Examination of the relationship between non-acute T1 and HRV has predominantly been in selected injured populations with the spinal cord, head/brain, and psychological trauma (post-traumatic stress disorder-PTSD and depression) [21, 22, 23, 24]. While systematic reviews have been conducted previously to assess the association between HRV and traumatic brain injury [25], spinal cord injury [26], PTSD [27] and depression [28], the longer-term effects of unselected TI on HRV have not been reviewed and warrant further examination [29]”.*

The above section highlights the lack of a systematic review on unselected TI and HRV- what this systematic review aims to address.

In the introduction, authors can also discuss what current guidelines indicate regarding the use of HRV indices in the management of non-acute injuries caused by trauma.

Response: Thanks for this suggestion. While we have adhered to the guidelines of the Task Force for reporting HRV indices throughout this systematic review, to the authors’ knowledge, there is no guideline on the use of HRV indices in the management of non-acute TI and trauma. As mentioned in the introduction section, several studies have explored the impact of acute trauma (immediately upon hospital admission) and selected TI (spinal cord/brain injury) on HRV, but no guidelines exist due to limited evidence on long-term HRV and non-acute TIs. This has been highlighted in the ‘future research section’ of our manuscript. Furthermore, at present, the use of HRV in non-acute traumatic injury is restricted to research studies. However, HRV has been widely adopted into decision-making and training protocols among athletes in everyday practice. With further research, we strongly believe that the use of HRV parameters will be integrated into clinical practice and for the rehabilitation of patients with a traumatic injury.

This sentence needs to be rewritten in the introduction: "What is the relationship between non-acute TI (E) and HRV (O) in people with unselected TI (P) compared to people who haven't been hurt (C)?"

Response: We appreciate this suggestion. However, the terms “people” and “hurt” may be too generic in terms of Population, exposure, control, and outcome (PECO) format. We would like to stick with the original sentence (lines 93-95): What is the association between non-acute TI *(E)* and HRV *(O)* in adults with unselected TI *(P)* versus uninjured controls *(C)*? because it focuses on a particular population (adults) and injury status (injured vs uninjured).

A few typing errors need to be reviewed.

Response: Thanks for highlighting this. The manuscript has now been proofread for typing/grammatical errors and the changes have been made in both (tracked and untracked) versions of the manuscript.

In the limitations section, authors should also discuss that non-linear HRV indices were not included in this study owing to the limited availability of data and that additional research is required to evaluate the effect of non-acute TI on standard HRV indices in civilian and military individuals separately.

Response: Thank you for this suggestion. The above sentences have been included in the limitation (lines 343-344) and future research sections (lines 358-359), respectively.
